# Isolation of a novel amylase and lipase-producing *Pseudomonas luteola* strain: study of amylase production conditions

**DOI:** 10.1186/1476-511X-13-9

**Published:** 2014-01-09

**Authors:** Lamia Khannous, Mouna Jrad, Mouna Dammak, Ramzi Miladi, Nour Chaaben, Bassem Khemakhem, Néji Gharsallah, Imen Fendri

**Affiliations:** 1 Faculté des Sciences de Sfax, Unité de recherche Toxicologie- Microbiologie Environnementale et Santé, Université de, Sfax, Tunisia; 2 Laboratoire de Biotechnologie des Plantes, Faculté des Sciences de Sfax, Université de Sfax, Sfax, Tunisia; 3 Département de génie biologique, Ecole Nationale d’Ingénieurs de Sfax, Université de Sfax, Sfax, Tunisia

**Keywords:** *Pseudomonas luteola*, Amylase, Lipase, Box Behnken Design

## Abstract

An amylase and lipase producing bacterium (strain C2) was enriched and isolated from soil regularly contaminated with olive washing wastewater in Sfax, Tunisia. Cell was aerobic, mesophilic, Gram-negative, motile, non-sporulating bacterium, capable of growing optimally at pH 7 and 30°C and tolerated maximally 10% (W/V) NaCl. The predominant fatty acids were found to be C_18:1_ω7c (32.8%), C_16:1_ω7c (27.3%) and C_16:0_ (23.1%). Phylogenetic analysis of the 16S rRNA gene revealed that this strain belonging to the genus *Pseudomonas*. Strain C2 was found to be closely related to *Pseudomonas luteola* with more than 99% of similarity. Amylase optimization extraction was carried out using Box Behnken Design (BBD). Its maximal activity was found when the pH and temperature ranged from 5.5 to 6.5 and from 33 to 37°C, respectively. Under these conditions, amylase activity was found to be about 9.48 U/ml.

## Introduction

*Pseudomonas* species, ubiquitous in soil and water [1,2], are of considerable scientific and technological importance and comprise a taxon of metabolically versatile organisms capable of utilizing a wide range of simple and complex organic compounds [3]. During the last few years, *Pseudomonas* strains have been increasingly studied with increasing interest because of their importance in the fields of medicine, food technology, environmental microbiology and phytopathology [4]. They are known to be involved in the biodegradation of natural and toxic man-made chemical compounds. In addition, the bacterial genus *Pseudomonas* is a prolific producer of a number of extracellular enzymes, including lipase and amylase [5].

α-amylase (E.C 3.2.1.1) catalyses the hydrolysis of α-D-(1, 4) glycosidic linkages in starch and related carbohydrates. It is a key enzyme in the production of starch derivatives and also widely used in food, textile, paper, detergent, clinical, pharmaceutical and other industrial fields [6,7].

In the last decades, there has been lots of research on the amylases from animals [8], plants [9] terrestrial bacteria [2] and marine bacteria [6]. Interest in bacterial amylases has increased due to their biotechnological applications [10]. Many microorganisms such as bacteria, yeast, and fungi are known to secrete amylases during their growth on starch substrates, which makes starch derivates available to cells.

In conventional multifactor experiments, optimization of plant extraction is usually carried out by varying a single factor while keeping all other factors fixed at a specific set of conditions. It is not only time-consuming, but also usually incapable of reaching the true optimum due to ignoring the interactions among variables [11]. Response surface designs such as central composite design (CCD) and Box Behnken Design (BBD) are commonly selected for performing optimization studies. Compared to the CCD method, the BBD technique is considered the most suitable for evaluating quadratic response surfaces particularly in cases when predicting the response at the extreme level is not the goal of the model. The BBD technique is a three level design based upon the combination of two-level factorial and incomplete block designs [12,13]. The BBD method employs a spherical design with excellent predictability within the design space and it requires less experiment than the FFD or CCD with the same number of factors [14]. In addition, the BBD technique is rotatable or nearly rotatable regardless of the number of factors under consideration [14].

In our study, a new strain C2 was isolated from soil regularly contaminated with olive washing wastewater and the diversity of their extracellular hydrolytic enzymes (lipase and amylase) was studied. The results showed that stain C2, which was identified as *Pseudomonas luteola*, had a high amylase activity. Optimization extraction was carried out using Box Behnken Design (BBD). This amylase showed a high activity, lead to expect a great commercial value and a good prospect for industrial applications.

## Materials and methods

### Source of strains and media

Soil samples were collected in sterile bottles from soil regularly contaminated (for more than 3 years) with olive washing wastewaters in Sfax (Tunisia) and stored in the dark at 4°C until use. Ten strains were isolated. Strain C2, was used for further experiments. For this purpose, Luria-Berttani (LB) medium containing 10 g peptone, 5 g yeast extracts and 5 g NaCl per liter was used. Fifteen grams of agar per liter were added when solid medium was required. The pH was adjusted to 7.0 with 10 M of KOH solution. Aliquots of 25 ml were dispensed into flasks and sterilized by autoclaving them at 121°C for 20 min.

### Isolation of bacteria

Suspensions of 1% (w/v) soil sample from ground contaminated with industrial wastewater were incubated with the medium at 30°C under agitation at 180 rpm for 72 hours. The dominant microorganisms of this consortium were isolated by spreading 0.1 ml of tenfold serial solution on Petri dishes containing screening medium agar [2,15]. The various colonies were picked, isolated in pure cultures and then stored at -80°C. Among the strains isolated, the bacterial colony named strain C2 was used for further experiments.

### Strain characterization

The pH of the medium was adjusted with 5 M HCl or 10 M KOH (in 0.5-unit steps) to obtain pH values ranging between 4 and 12. Various amounts of NaCl were directly weighed in flasks prior to dispensing a 25 ml medium with the desired NaCl concentration (range from 0 to 250 g/l). The temperature range for growth was analyzed between 5 and 55°C (in 5°C intervals). Microscopes were performed as described by Abdelkafi *et al*. [16,17]. To test the heat resistance, cells grown in a basal medium containing olive oil were exposed to temperatures of 80, 90 and 100°C for 10 min. The cells were cooled quickly to ambient temperature and inoculated into fresh Luria broth medium, and their growth was recorded after 24 h incubation at 37°C under agitation at 180 rpm. The conditions under which sporulation was tested included growth in the absence of a carbon source or in the presence of yeast extract. Growth under anaerobic conditions was determined as described previously [18,19]. Gram reaction was determined using the BioMérieux Gram stain Kit according to the manufacturer’s instructions. Catalase activity was determined based on bubble production in a 3% (v/v) hydrogen-peroxide solution. Oxidase activity was determined by testing the oxidation of 1% *p-*aminodimethylaniline oxalate. Experiments were performed in duplicate with an inoculum subcultured at least once under the same test conditions. The substrates tested for utilization were injected from pre-sterilized and concentrated stock solutions into flasks containing a 25 ml pre-sterilized medium. The following substrates were used: carbohydrates (20 mM) (glucose, fructose, galactose, maltose and lactose); gelatin, peptone, and yeast extract (2 g/l) and glycerol (20 mM). An increase in the OD_600_ value obtained with substrate-containing cultures, compared with control tubes lacking substrates, was considered as positive growth. Other phenotypic characteristics were determined using API 20NE and API 50CH kits (BioMérieux, La Balme-Les-Grottes, France), as described by Logan and Berkeley [20]. In addition, the API ZYM gallery (BioMérieux, La Balme-Les-Grottes, France) method was used to determine the extracellular enzymatic activities.

Resistance to antibiotics was determined on Mueller–Hinton agar (Difco, Beckton Dickinson, Le Pont de Claix, France) using standard antibiotic disks (BioMérieux, Marcy l’Etoile, France). Inhibition diameters were recorded after 24 h of incubation at 30°C under aerobic conditions. Strains were classified as sensitive, not sensitive or intermediately sensitive to the antibiotics tested in the line with the disk manufacturer’s instructions. All tests were performed in triplicate.

### Analysis of cellular fatty acids

Fatty acid methyl esters were determined as described by Ben Ali et al. [19].

### **S**equencing and phylogenetic analysis

The 16S rRNA gene of strain C2 was amplified by adding 1-μL cell culture to a thermocycler microtube containing 5 μl of 10 × *Taq* buffer, 0.5 μl of each 50 nM primers Fd1 and Rd1, 5 μl of 25 mM MgCl_2_·6 H_2_O, 0.5 μl of 25 mM dNTPs, 0.5 μl of *Taq* polymerase (5 U/μl) and 38 μl of sterilized distilled water. The universal primers Fd1 and Rd1 (Fd1, 5′-AGAGTTTGATCCTGGCTCAG-3; Rd1, 5′-AAGGAGGTGATCCAGCC-3′) were used to obtain a PCR product with a molecular weight of ~1.5 kb corresponding to base positions 8 to 1542, based on *Escherichia coli* numbering of the 16S rRNA [21]. Each sample was placed in a hybrid thermal reactor thermocycler (BIOMetra), denatured by heating for 1 min at 96°C and subjected to 30 cycles for 20 s at 96°C, 30 s at 55°C and 2 min at 72°C. This was followed by a final 5-min elongation step at 72°C. PCR products were cloned using the pGEM-T-easy cloning kit (Promega) as recommended by the manufacturer. Clone libraries were screened by performing direct PCR amplification on a colony using the vector-specific primers SP6 (5′-ATTTAGGTGACACTATAGAA-3′) and T7 (5′-TAATACGACTCACTATAGGG-3′) and the following reaction conditions: initial 2-min denaturation at 96°C, then 40 denaturation cycles, annealing and extension for 30 s at 96°C, 30 s at 50°C, 2 min at 72°C, and a final extension for 5 min at 72°C. Plasmids containing inserts of the expected length were isolated using the Wizard Plus SV Minipreps DNA purification system (Promega), as recommended by the manufacturer. Purified plasmids were sent for sequencing to GATC Company (France). Sequence data was imported into the sequence editor BioEdit version 5.0.9 [22]; base calling was examined and a contiguous sequence was obtained. The full sequence was aligned using the RDP Sequence Aligner program [23]. The consensus sequence was adjusted manually to fit the 16S rRNA secondary structure model [21]. A non-redundant BLAST search [24] was performed to identify the closest relatives. Sequences used in the phylogenetic analysis were obtained from the RDP [23] and GenBank databases [25]. Positions of sequence and alignment ambiguities were omitted and pairwise evolutionary distances were calculated using the method of Jukes and Cantor [26]. A dendrogram was constructed using the neighbor-joining method [27]. Confidence in the tree topology was determined using 100-bootstrapped trees [28].

### Lipid utilization and lipase activity assay

To study the ability of the new isolate to grow on lipids, experiments were performed on agar plates with rhodamin B and in liquid cell cultures with and without a lipid carbon source. To analyse the ability of this strain to grow and degrade lipids, filtered olive oil was added to the medium (2%, v/v) [29]. Lipase activity was assayed as described by Abdelkafi et al. [30,31].

### Amylase activity assay

The amount of the reducing sugars released by the action of amylases on starch was currently performed at 60°C and pH 5 for 10 min [7]. The reaction mixture contained 0.5% (w/v) starch in 25 mM acetate buffer and the enzyme solution in a final volume of 1 ml. The concentration of reducing sugar was determined by the DNS method [32]. One unit of amylase was defined as the amount of enzyme, required to produce reducing sugars equivalent to 1 μmol glucose/min.

### Effect of temperature and pH

The effect of temperature on the activity of extracellular amylase was performed by its supernatant incubation at different temperatures ranging from 40 to 100°C. The reaction was performed according to the method of amylase assays described above. The effect of pH on the activity of amylase was determined at pH ranging from 4 to 10.5 at 60°C. The buffers used are: sodium acetate 0.1 M (pH 4–6.5), phosphate 0.1 M (pH 7–9) and glycine-NaOH 0.1 M (pH 9–10.5). The activities at optimal temperature and pH were defined as 100%.

### Thermal stability

Amylases in sodium acetate buffer (50 mM, pH 5.0) were incubated at a temperature range of 50–60°C and then samples were withdrawn for enzyme assay at appropriate time intervals. The residual activity was estimated taking original activity as 100% [7].

### pH stability

Stability of amylases was determined using different buffers such as glycine–HCl (pH 2.0–3.0), sodium acetate (pH 4.0–6.5), sodium phosphate (pH 7.0–8.0), glycine–NaOH 0.1 M (pH 9–10.5) at 50 mM concentration. Amylase was incubated in different buffers for 24 h at room temperature. The original activity was taken as control (100%) [7].

### Experimental design and data analysis

The Box Behnken Design (BBD) composite, with three replicates at the central points was employed to fit a second-order polynomial model and to obtain an experimental error. The BBD was applied with three design factors and three levels. The factors are the medium pH (X1), the temperature (X2) and the time of contact between substrate-enzyme (X3). The coded levels and the natural values of the factors set in this statistical experiment are shown in Table 1. The central values (zero level) chosen for the experimental design were as follows: the pH, 7; the temperature, 37°C and the contact time, 24 h. The substrate chosen in this study was the starch.

**Table 1:** Levels of the variables tested in the BBD designs

**Variables**	**Range and levels**		
	**-1**	**0**	**1**
**X1, pH**	4	7	10
**X2, Temperature (°C)**	30	37	44
**X3, Incubation time (h)**	12	24	36

Amylase activity was selected as the dependent variable. The response variable was fitted by a second-order model in the form of quadratic polynomial equation:

$$y=b_{0}+\sum_{i=1}^{k}b_{i}X_{i}+\sum_{i=1}^{k}b_{\mathrm{ii}}X_{i}+\sum_{i}^{i<j}\sum_{j}b_{\mathrm{ij}}X_{i}X_{j}$$

where y is the response variable to be modeled; Xi, and Xj the independent variables which influence y; b_0_, b_i_, b_ii_ and b_ij_ are the offset terms, the *ith* linear coefficient, the quadratic coefficient and the *ijth* interaction coefficient, respectively. The actual design used in this work is presented in Table 2. Analysis of variance (ANOVA) was used for graphical analyses of the data to obtain the interaction between the process variables and the responses. The quality of the fit polynomial model was expressed by the coefficient of determination R^2^, and its statistical significance was checked by the *F*-test. Model terms were selected or rejected based on the p-value (probability) with 95% confidence level. Three-dimensional plots and their respective contour plots were obtained based on the effects of the levels of two factors. The experimental design, regression and statistical analysis were performed, by NemrodW® software [33]. The optimum values of selected variables were obtained by using the desirability function available in NemrodW® and also by analyzing the response surface contour plots. The parameters of the response equations and corresponding analysis on variations were evaluated using Uniform Design Software 2.1 (http://en.softonic.com/s/uniform-design-software-2.1) and MATLAB 6.5, respectively. The interactive effects of the independent variables on the dependent ones were illustrated by three and two-dimensional contour plots. Finally, two additional experiments were conducted to verify the validity of the statistical experimental strategies.

**Table 2:** BBD and response results

**Runs**	**Factors**			**Response: amylase activity (U/ml)**
	**X1**	**X2**	**X3**	
1	0	0	0	9.48
2	1	-1	0	4.49
3	0	0	0	9.14
4	-1	0	1	1.7
5	-1	0	-1	7.08
6	0	0	0	9.42
7	0	-1	1	1.8
8	0	0	0	8.97
9	0	1	1	4.48
10	-1	0	-1	5.45
11	0	-1	-1	8
12	-1	0	1	8.31
13	0	1	-1	3.65
14	-1	-1	0	8.68
15	-1	1	0	5.97

## Results and discussion

### Isolation of bacteria

To isolate different lipid-degrading microorganisms, an enrichment culture method was used as described in Materials and Methods. Based on their morphological characteristics, several strains were isolated. The strain used in this work, named C2, was selected for further characterization. Species belonging to the genus *Pseudomonas* have been isolated from a variety of natural sources including soil, plants, marine environments, and activated sludge [34,35]. The present results extended the range of known ecosystems to include soil contaminated with olive washing wastewater.

### Morphology and physiology

The organism used in this study was a mesophilic strain which was isolated from soil contaminated with wastewater. Cells of strain C2 were Gram-negative, motile and aerobic. Similar characteristics have been described in other *Pseudomonas* species by [4,35,36]. Spores were not observed.

The optimum pH and temperature values for the growth of this isolate were first determined under aerobic conditions in Erlenmeyer flasks. Growth was observed at temperatures ranging between 20 and 50°C, and optimum growth occurred at 30°C, but the isolate did not grow at 56°C. The pH range for growth was 5–9, with an optimum at 7. Growth occurred in the 0 to 10% NaCl range. Anaerobic growth does not occur. Catalase and oxidase reactions were positive and negative, respectively. Citrate was used. Gelatine was hydrolyzed. Other characteristics of strain C2 in comparison with those of the closely related species of genus *Pseudomonas* are given in Table 3.

**Table 3:** **Main characteristics of strain C2 and *Pseudomonas luteola***

**Characteristics**	**Strain C2**	***Pseudomonas luteola***
Gram stain	─	─
Catalase	+	+
Oxidase	─	─
Motility	+	+
Colour	nd	+
Optimum temperature growth (°C)	30	30
Growth at 40°C	+	+
Growth with 8% NaCl	+	─
Optimum pH growth	7	7.2
Denitrification	─	─
Gelatinase	+	+
Amylase	+	nd
**Assimilation of:**		
Citrate	**+**	+
Malate	**+**	+
Maltose	+	+
Mannitol	**+**	+
Mannose	**+**	+
Glucose	─	+

+, positive; ─, negative; nd, not determined.

### Properties of strain C2 in API-ZYM test

The strain under investigation showed a high rate of enzyme activity for alkaline phosphatase, esterase (C4), esterase lipase (C8), leucine arylamidase, trypsin, acid phosphatase and cystine arylamidase. No activity was detected for α-mannosidase and α-fucosidase.

### Antibiotic susceptibility

The growth behaviour of the C2 isolate was studied in the presence of a range of antibiotics. The strain was susceptible to penicillin (6 μg), chloramphenicol (30 μg), kanamycin (30 IU) and tetracycline (30 IU); and resistant only to erythromycin (15 μg).

### Fatty acid composition

The cellular-fatty-acid profiles of strain C2 show the presence of large amounts of saturated and unsatured fatty acids (Table 4). The main fatty acids detected in this strain were C_18:1_ω7c (32.8%), C_16:1_ω7c (27.3%) and C_16:0_ (23.1%), which together accounted for >83% of the total fatty acids. Similar fatty acid components predominate in other species of the genus *Pseudomonas* such as *Pseudomonas xiamenensis*[34]. These results confirming that this strain belongs to the genus *Pseudomonas*. However, some considerable differences between the fatty acid profiles were detected. For example, C_17:0_ cyclo, detected in other *Pseudomonas* strains such as *Pseudomonas simiae* CCUG 50988^T^ (11.7%) [37]. This discrepancy may have resulted from differences between the experimental conditions used, in the growth conditions or the analytical equipment used, for example. Other minor components present included C_10:0_ 3-OH (3.4%), C_12:0_ (5.2%), C_12:0_ 2-OH (3.3%), C_12:0_ 3-OH (2.1%), C_14:0_ (0.8%) and C_18:0_ (0.4%).

**Table 4:** **Cellular fatty-acid profiles (%) of *Pseudomonas *** **sp. strain C2 in comparison with those of *Pseudomonas luteola *** **IAM 13000**^**T**^

**Fatty acids**	**Strain C2**	***P. luteola***
**C**_**10:0**_**3OH**	3.4	3.8
**C**_**12:0**_	5.2	6.9
**C**_**12:0**_**2OH**	3.3	3.2
**C**_**12:0**_**3OH**	2.1	3.5
**C**_**14:0**_	0.8	1.0
**C**_**16:0**_	23.1	18
**C**_**18:0**_	0.4	0.2
**C**_**19:0**_**CYCLO** ω**8c**	ND	0.3
**C**_**16:1**_ω**7c**	27.3	23.3
**C**_**18:1**_ω**7c**	32.8	39.7

ND, not detected.

### Phylogenetic analysis

**Figure 1:**
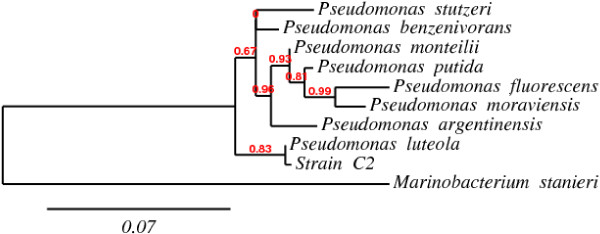
**Phylogenetic dendrogram based on 1247 unambiguous bp of the 16S rRNA sequences indicating the position of *Pseudomonas luteola *** **strain C2 and its closest relative sequences validated at species level in the genus *Pseudomonas*.**

To analyze the phylogenetic position of C2, the 16S rRNA gene sequence (comprising 1378 bases) was determined and a phylogenetic tree based on 1247 unambiguous bases was constructed (Figure 1). Phylogenetic analysis showed that strain C2 is a member of the *Gammaproteobacteria*. Dendrogram analysis showed that this isolate formed a coherent cluster with species of the genus *Pseudomonas*. Our isolate was found to be closely related to *Pseudomonas luteola*, with which it shows more than 99% similarity between the 16S rRNA gene sequences.

### Lipid degradation

Agar plates containing olive oil and rhodamine B have a pinkish colour and an opaque appearance. Lipase production was monitored by irradiating plates with UV light at 350 nm. After 24 h of incubation, bacterial colonies began to show orange fluorescence; with longer incubation times, orange fluorescent halos were formed around the colonies of lipase producing strains. Strain C2 showed fluorescent halo (data not shown). It has been established that *Pseudomonas* species can degrade various triacylglycerol [38,39].

### Optimization of amylase extraction

The effect of the temperature and the pH on the amylase activity in the supernatant was performed by its incubation at different temperatures ranging from 40 to 100°C and at pH ranging from 4 to 10.5. The highest amylase activity was given at 60°C and at pH 5 (data not shown). In this work, the Box Behnken design (BBD) was used to study the effects of the three variables chosen towards their response and subsequently in the optimization study. Experiments according to the design were carried out and relevant results are shown in Table 2, which lists amylase activity. The mathematical equation given by the model is:

$$\begin{array}{c} \mathrm{Amylase}\;\mathrm{activity}\left(U/\mathrm{ml}\right)=9.207–0.426\;X_{1}-0.598\;X_{2}\\ -1.389\;X_{3}–1.413\;{X_{1}}^{2}–1.761\;{X_{2}}^{2}–2.963\;{X_{3}}^{2}\\ +0.577\;X_{1}X_{2}+1.255\;X_{1}X_{3}+1.758\;X_{2}X_{3} \end{array}$$

**Figure 2:**
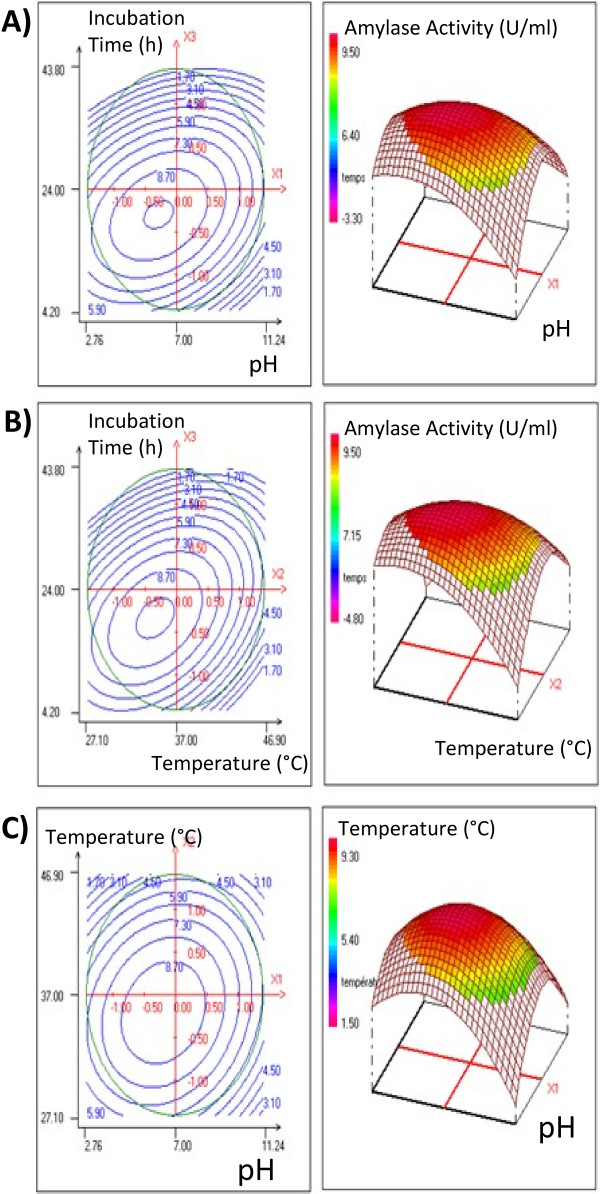
Surface graphs of response Y (amylase activity U/ml) showing the effects of variables: A, effect of the incubation time versus pH; B, effect of the incubation time versus temperature; C, effect of the temperature versus pH.

The results obtained are then analyzed by “F” statistical test for analysis of variance (ANOVA) to assess the “goodness of fit” (Table 5). The model equation adequately describes the response surfaces of Amylase activity in the interval of investigation. The model is found to be significant at 95% confidence level by the *F*-test as shown in Table 5, with all *p*-values of regression ≤0.05. With *Pseudomonas luteola* amylase activity as the response, the response surface (3D) and the contour plots (2D) of the quadratic model were shown in Figure 2. The obvious trough in the response surfaces indicates that the optimal conditions were exactly located inside the design boundary.

**Table 5:** Statistical parameters obtained from the ANOVA test performed for the model

**Source of variation**	**Sum of square (SS)**	**Degree of freedom (ddl)**	**Average square**	**Fisher number**	**Signification**	**R**^**2**^
**Regression**	85.2004	9	9.4667	155.3619	0.641**	0.93
**Residues**	6.4013	5	1.2803	-	-	-

**p-value < 0.01.

The surface graphs of response (amylase activity) showed a considerable curvature in contour curves, implying that these three factors were interdependent (Figure 2). There were significant interactive effects on amylase activity between temperature (°C), pH and incubation time (h). The contour plot of Temperature versus pH showed that the optimal conditions for the response were located in the region, where temperature ranged from 32 to 38°C and pH from 5.5 to 7.5 (Figure 2C). The contour plots of incubation time versus pH or temperature showed that the optimal conditions for the response were obtained after 19–22 h of incubation and when pH and temperature ranged from 5.5 to 6.5 and from 33 to 37°C, respectively (Figure 2A and 2B).

Under these conditions, the C2 amylase activity can reach 9.48 U/ml. Similar results were obtained with *Lactobacillus pentosus* N3 and *Lactobacillus plantarum* Bom 816 when amylase activity ranged from 3–4 to 21 U/ml and from 0.5 to 11.5 U/ml, respectively [40].

## Conclusion

A strain isolated from a sample of soil was selected for its ability to produce amylase. This isolate was identified by 16S rRNA gene sequences analysis as *P. luteola*. Amylase optimization extraction was carried out using Box Behnken Design (BBD). The effect of three really influencing variables: temperature (°C), pH and incubation time (h) on amylase activity was investigated. Its maximal activity was in the pH and temperature ranged from 5.5 to 6.5 and from 33 to 37°C, respectively. Under these conditions, the C2 amylase activity can reach 9.48 U/ml. The cloning and expression of its amylase is under current investigation.

## Competing interests

The authors declare that they have no competing interests.

## Authors’ contributions

LK, MJ, NC, MD, RM and MB performed the experiments. LK, NG and IF designed the experiments, analyzed the data and conceived research. LK drafted the manuscript. LK, NG and IF have approved the final version of the manuscript. All authors read and approve the final manuscript.
